# Editorial: Inflammation and immunomodulation in cardiovascular remodeling

**DOI:** 10.3389/fcvm.2022.1094094

**Published:** 2022-12-16

**Authors:** Mark Ewing, Jacco C. Karper, Margreet R. de Vries, Paul H. A. Quax

**Affiliations:** ^1^Department of Cardiology, Noordwest Ziekenhuisgroep (NWZ), Alkmaar, Netherlands; ^2^Department of Cardiology, Wilhelmina Ziekenhuis Assen (WZA), Assen, Netherlands; ^3^Einthoven Laboratory for Experimental Vascular Medicine, Department of Surgery, Leiden University Medical Center, Leiden, Netherlands

**Keywords:** inflammation, immunomodulating, editorial, cardiovascular, remodeling

This editorial features the collection of articles published in Frontiers in Cardiovascular Medicine: *Inflammation and Immunomodulation in Cardiovascular Remodeling*. The goal of this Research Topic is to focus on the different components of the immune system that can potentially influence the cardiovascular remodeling process. In addition, it is important how new insights such as gender differences and the influence of the microbiome may play a role. It is important to look for new therapies or strategies that exert their work through immunomodulation. And immunomodulation is an exciting field in regard to developing novel therapies and strategies. Following are the original research papers and (systematic) reviews published in this issue in 2021–2022 (Table 1).

**Table 1:** Metrics (on October 22nd 2022) of the highlight's articles published in Frontiers in Cardiovascular Medicine, Inflammation, and Immunomodulation in cardiovascular remodeling, in 2021–2022.

**Title**	**First author, country**	**Views**	**Downloads**
Bioinformatics and Immune Infiltration Analyses Reveal Key Pathway and Immune Cells in The Pathogenesis of Hypertrophic Cardiomyopathy	Xu-Zhe Zhang, China	3,824	1,513
Sex Differences in Inflammation during Venous Remodeling of Arteriovenous Fistulae	Shin Mei Chan, United States	1,622	397
Molecular Interactions between Vascular Smooth Muscle Cells and Macrophages in Atherosclerosis	Stephanie Lehoux, Canada	2,395	598
Stimulation of the PD-1 pathway decreases atherosclerotic lesion development in Ldlr deficient mice	Hendrika W. Grievink, The Netherlands	1,337	474
Association of neutrophil to lymphocyte ratio with plaque rupture in acute coronary syndrome patients with only intermediate coronary artery lesions assessed by optical coherence tomography	Huili Zhang, China	1,477	187
Interfering in the ALK1 pathway results in macrophage-driven outward remodeling of murine vein grafts.	Alwin de Jong, The Netherlands	1,696	424
The intriguing role of TLR accessory molecules in cardiovascular health and disease	Taisiya Bezhaeva, The Netherlands	2,738	928
Inflammatory mediators in atherosclerotic vascular remodeling	Bryce Evans, Switzerland	1,825	350
Identifying novel mechanisms of abdominal aortic aneurysm *via* unbiased proteomics and systems biology	Stephanie Morgan, United States	1,454	319

*Bioinformatics and Immune Infiltration Analyses Reveal Key Pathway and Immune Cells in The Pathogenesis of Hypertrophic Cardiomyopathy* by Zhang et al. This study was designed to identify key pathway and immune cells for hypertrophic cardiomyopathy (HCM) *via* bioinformatics analyses of public datasets and evaluate the significance of immune infiltration in the pathogenesis of HCM. They found that the STAT3-related pathway and CD163+LYVE1+ macrophages were identified as the potential key pathway and immune cells in HCM and may serve as interesting targets for further deepening research.

*Sex Differences in Inflammation during Venous Remodeling of Arteriovenous Fistulae* by Chan et al. Vascular disorders frequently have differing clinical presentations among women and men. Sex differences exist in vascular access for hemodialysis; women have reduced rates of arteriovenous fistula (AVF) maturation as well as fistula utilization compared with men. Inflammation is increasingly implicated as a potent mechanism driving AVF maturation, especially in vessel dilation and wall thickening, allowing venous remodeling to support hemodialysis. Here, they review the current knowledge and describe several crucial regulators of remodeling such as ephrins, T cells and macrophages and especially downstream genes are affected by sex differences and female hormones.

*Molecular Interactions between Vascular Smooth Muscle Cells and Macrophages in Atherosclerosis* by Beck-Joseph and Lehoux et al. Vascular smooth muscle cells and macrophages play dominant roles in atherosclerosis. A firm understanding of how these cells influence and modulate each other is pivotal for a better understanding of the disease and the development of novel therapeutics. Recent studies have investigated molecular interactions between both cell types and their impact on disease progression. Here, Intercellular communications through soluble factors, physical contact, and extracellular vesicles are discussed and their impact on atherosclerotic lesion formation is reviewed with respect to the current knowledge.

*Stimulation of the PD-1 pathway decreases atherosclerotic lesion development in Ldlr deficient mice* by Grievink et al. Signaling through the coinhibitory programmed death (PD)-1/PD-Ligand 1 pathway regulates T cell responses and can inhibit ongoing immune responses. Dampening the excessive immune response by promoting PD-1/PD-L1 signaling may have a high therapeutic potential to limit disease burden. With the current and increasing use of immune check point inhibition as cancer therapies, including inhibition of the PD-1/PD-L1 signaling, this is a very interesting point. This study aimed to assess whether an agonistic PD-1 antibody can diminish atherosclerosis development. They showed that stimulation of the coinhibitory PD-1 pathway inhibits atherosclerosis development by modulation of T- and B cell responses. These data support stimulation of coinhibitory pathways as a potential therapeutic strategy to combat atherosclerosis.

*Association of neutrophil to lymphocyte ratio with plaque rupture in acute coronary syndrome patients with only intermediate coronary artery lesions assessed by optical coherence tomography* by Jiang et al. Plaque vulnerability and rupture rather than plaque size are the major cause of clinical events in patients with intermediate coronary lesions. Therefore, the present study was aimed to explore potential markers associated with plaque rupture in acute coronary syndrome patients with intermediate coronary lesions. They found that the neutrophil to lymphocyte ratio (NLR) biomarker is closely associated with plaque rupture. Monitoring NLR may be useful in risk stratification and management for intermediate coronary artery lesions.

*Interfering in the ALK1 pathway results in macrophage-driven outward remodeling of murine vein grafts* by Jong et al. Vein grafts are frequently used to bypass coronary artery occlusions. Unfortunately, vein graft disease (VGD) causes impaired patency rates. ALK1 mediates signaling by TGF-β *via* TGFβR2 or BMP9/10 *via* BMPR2, which is an important pathway in fibrotic, inflammatory, and angiogenic processes in vascular diseases. A role of the TGF-β pathway in VGD is previously reported, however, the contribution of ALK1 signaling was unknown. They investigated ALK1 signaling in VGD in a mouse model for vein graft disease using either genetic or pharmacological inhibition of the Alk1 signaling and found that reduced ALK1 signaling in VGD promotes outward remodeling, increases macrophage influx and promotes an unstable plaque phenotype.

*The intriguing role of TLR accessory molecules in cardiovascular health and disease* by Bezhaeva et al. The present review outlines accessory molecules for membrane TLRs that are involved in cardiovascular disease progression. They summarize the up-to-date knowledge on TLR signaling focusing on membrane TLRs and their ligands that play a key role in cardiovascular system. They then survey the current evidence of the contribution of TLRs accessory molecules in vascular and cardiac remodeling including myocardial infarction, heart failure, stroke, atherosclerosis, vein graft disease and arterio-venous fistula failure.

*Identifying novel mechanisms of abdominal aortic aneurysm via unbiased proteomics and systems biology* by Morgan et al. Abdominal aortic aneurysm (AAA) leads to rupture if not surgically repaired. Mice aid the study of disease progression and its underlying mechanisms. The present study used unbiased proteomics and systems biology to understand the molecular relationship between the mouse models of AAA and the human disease. Aortic tissues of developing and established aneurysms produced by either angiotensin II (AngII) infusion in Apoe -/- and Ldlr -/- mice or intraluminal elastase incubation in wildtype C57BL/6J mice were examined as well as human samples of infrarenal aortic aneurysm tissues and aortic tissue collected from age-matched controls. They found that the aneurysmal tissue from both mouse and human had inflammation, coagulation, and protein processing signatures, but differed in the prevalence of neutrophil-associated pathways, and erythrocyte and oxidative stress-dominated networks in the human aneurysms. Moreover, the conclude that identifying changes unique to each mouse model will help to contextualize model-specific findings. Focusing on shared proteins between mouse experimental models or between mouse and human tissues may help to better understand the mechanisms for AAA and establish molecular bases for novel therapies.

*Inflammatory mediators in atherosclerotic vascular remodeling* by Evans et al. Atherosclerosis is initiated by endothelial dysfunction allowing the accumulation of intimal lipids and leukocytes. Inflammatory mediators such as cytokines, chemokines, and modified lipids further drive vascular remodeling ultimately leading to thrombus formation and/or vessel occlusion which can cause major cardiovascular events. Although it is clear that vascular wall remodeling is an elementary mechanism of atherosclerotic vascular disease, the diverse underlying pathophysiological mechanisms and its consequences are still insufficiently understood. Here, they review the current knowledge on the involvement of the various Chemokines and Cytokines, as well as other inflammatory and immune-modulating factors in the development and progression of atherosclerosis and what the role of these key inflammation driving factors in the various cell types in the vessel wall is.

The above presented collection of papers demonstrates the broad involvement of the immune system in cardiovascular remodeling. It is also highlights the importance of specific population differences such as gender. The selected reviews provide a clear structural overview of the current knowledge of the involvement of, for example, TLR accessory molecules, inflammatory mediators in atherosclerosis and interaction between macrophages and smooth muscle cells. In addition, there is innovative research that helps us understanding aneurysm formation and the influence of the chosen animal model on previously reported results. Other papers show new pathways in cardiovascular remodeling thereby exploring new potential therapeutic targets.

In our view, this collection of papers provides an interesting overview of how much the immune system is involved in the pathophysiological process of cardiovascular remodeling. This makes the immune system a potential therapeutic target for developing new interventions and clinical data seems to confirm this. Translational models are very useful but of course also have limitations. Knowledge and understanding of the involvement of the immune system is highly desirable given the rapid increase in specific immune modulation therapies in other fields such as rheumatology and cancer treatment. Without a doubt this has been a game changer for many patients, but we need to be vigilant about the potential adverse effects. Exploring pathophysiological mechanisms understanding involvement and interactions regarding immune processes is therefore crucial.

## Author contributions

JK, MV, and PQ: writing–review and editing. ME: writing–original draft. All authors contributed to the article and approved the submitted version.

